# Multipotential Alkaline Protease From a Novel *Pyxidicoccus* sp. 252: Ecofriendly Replacement to Various Chemical Processes

**DOI:** 10.3389/fmicb.2021.722719

**Published:** 2021-10-11

**Authors:** Sonia Sharma, Shiv Kumar, Rajinder Kaur, Ramandeep Kaur

**Affiliations:** ^1^Department of Biotechnology, Guru Nanak Dev University, Amritsar, India; ^2^Department of Botanical and Environmental Sciences, Guru Nanak Dev University, Amritsar, India; ^3^Department Cum National Centre for Human Genome Studies and Research, Panjab University, Chandigarh, India

**Keywords:** *Pyxidicoccus* sp., myxobacteria, alkaline proteases, surfactant and oxidant stable proteases, chitin extraction, bioextraction

## Abstract

A newly isolated alkaline protease-producing myxobacterium was isolated from soil. The strain was identified as *Pyxidicoccus* sp. S252 on the basis of 16S rRNA sequence analysis. The extracellular alkaline proteases produced by isolate S252 (PyCP) was optimally active in the pH range of 11.0–12.0 and temperature range of 40–50°C The zymogram of PyCP showed six caseinolytic protease bands. The proteases were stable in the pH range of 8.0–10.0 and temperature range of 40–50°C. The activity of PyCP was enhanced in the presence of Na^+^, Mg^2+^, Cu^2+^, Tween-20, and hydrogen peroxide (H_2_O_2_) (hydrogen peroxide), whereas in Triton X-100, glycerol, ethylenediaminetetraacetic acid (EDTA), and Co^2+^, it was stable. PyCP showed a potential in various applications. The addition of PyCP in the commercial detergent enhanced the wash performance of the detergent by efficiently removing the stains of tomato ketchup and coffee. PyCP efficiently hydrolyzed the gelatin layer on X-ray film to release the embedded silver. PyCP also showed potent dehairing of goat skin and also efficiently deproteinized sea shell waste indicating its application in chitin extraction. Thus, the results of the present study indicate that *Pyxidicoccus* sp. S252 proteases have the potential to be used as an ecofriendly replacement of chemicals in several industrial processes.

## Introduction

Proteases are hydrolytic enzymes that are capable of catalyzing the hydrolysis of peptide bonds and thus degrade proteins into simpler peptides. Proteases can be classified based on the site of action, substrate specificity, similarity to studied enzymes, amino acids at the active site, and pH optima (Banerjee and Ray, 2017). On the basis of the site of action, they are categorized into endo- or exo-enzymes, and based on the amino acids at the active site, they are classified as serine, cysteine, aspartic, and metallo proteases. Based on the pH optima, they are classified as acidic, neutral, and alkaline proteases (Rao et al., 1998). A comprehensive compilation of proteases can be accessed in the MEROPS peptidase database (Rawlings et al., 2018).

Proteases are ubiquitous in prokaryotes and eukaryotes, where they play important roles in physiology of the host organisms. Proteases are also commercially important and command the largest share (60%) of the enzymes used in industrial processes. Alkaline proteases account for 35% of these proteases and are used in food, leather, silk, laundry, tannery, cosmetics, and pharmaceutical industries (Ramkumar et al., 2018). Furthermore, microorganisms are the preferred source for industrial enzymes because they are easy to maintain and cultivate and are amenable to genetic manipulations enabling higher yield of the enzymes (Asgher et al., 2018; Zhou et al., 2018; Yu et al., 2019).

The enzymes are environmentally safe substitutes for hazardous chemicals in industrial processes (Razzaq et al., 2019). The enzymes for industrial use are produced in bulk and can be employed as crude preparations to make the process more cost effective. The use of crude proteases suffices for most applications such as, in detergents, bioactive protein hydrolysate preparation, bioextraction of chitin from shrimp shells, hide dehairing, and silver recovery from X-ray film (Cavello et al., 2013; Paul et al., 2014; Hammami et al., 2017, 2018; Murthy et al., 2018). The need to resort to the expensive and time-consuming process of purification of proteases is limited to their use in pharmaceutical and medical applications (Banerjee and Ray, 2017). A pre-requisite for an enzyme to be successfully adopted in industries is that it should have the ability to withstand harsh process conditions viz. temperature, pH, and presence of inhibitors. The diversity of microbes inhabiting the planet enables the discovery of enzymes with the desirable industrial features.

Myxobacteria are Gram-negative, unicellular, non-pathogenic δ-proteobacteria, and they inhabit soil, decaying plant material, bark of trees, herbivore dung, and the marine environment (Reichenbach and Dworkin, 1992; Dawid, 2000; Iizuka et al., 2013; Garcia et al., 2014). They are generally considered mesophiles (6–38°C) and grow in wide pH range of 5–9. Myxobacteria show cooperative behavior, as is evident from the swarming of cells which is the result of cell-cell communication and cooperation among the cells (Dawid, 2000). Myxobacteria practice group predation and produce a wide variety of hydrolytic enzymes to lyse the prey cells for acquiring nutrients (Muñoz-Dorado et al., 2016). To sustain in the competitive environmental niche, it is expected that myxobacteria would secrete robust and efficient proteases which can withstand harsh environmental conditions. With the growing emphasis of industrial processes toward cleaner technologies, the attention is focused on the discovery of robust enzymes with unique properties. Though myxobacteria have been extensively explored for bioactive secondary metabolites, there are very scant reports of the studies on the industrially important enzymes from myxobacteria. Recently, prolyl oligopeptidase from *Myxococcus xanthus* that had shown promise in the preparation of bioactive peptides by gluten hydrolysis was expressed in *Escherichia coli* (Shan et al., 2004; Kocadag Kocazorbaz and Zihnioglu, 2017). A novel milk extracellular clotting protease with utility in cheese industry was characterized from *M. xanthus* strain 422 (Poza et al., 2003).

In view of the emphasis on making the industrial processes ecologically sustainable, the present study aimed to determine the biotechnological potential of proteases produced by the newly isolated strain of myxobacterium, *Pyxidicoccus* sp. S252. The application of the extracellular proteases produced by isolate S252 (PyCP) as detergent additive, in hydrolysis of gelatin for silver recovery from used X-ray films and animal hide dehairing was evaluated. To the best of our knowledge, this is the first report on the characterization and potential applications of alkaline proteases from a myxobacterium of the genus *Pyxidicoccus*.

## Materials and Methods

### Isolation and Identification of S252

Myxobacteria were isolated as described in Kumar et al. (2017). Briefly, soil samples were placed on *E. coli* paste spread on water calcium chloride clerigel (WCX clerigel; 0.1% CaCl_2_⋅2H_2_O, 20 mM HEPES buffer, 0.8% clerigel, and 50 μg/ml cycloheximide; pH 7.2). The strains were purified by picking cells from the edge of the swarm that emerged from soil sample and were grown on casitone yeast (CY) agar medium (0.3% casitone, 0.1% yeast extract, 0.1% CaCl_2_⋅2H_2_O, and 1.5% agar; pH 7.2) for growth. The isolated bacterial strains were subjected to Gram staining. The ability of the myxobacterial strains to produce extracellular protease was tested on skim milk agar (1% skim milk, 0.1% CaCl_2_⋅2H_2_O, 2% agar, pH 11).

Genomic DNA was isolated from the cells scrapped from freshly streaked and overnight incubated culture plates using genomic DNA isolation kit (RBC, Taiwan). The genomic DNA was used as template to amplify 16S rRNA gene using the universal bacterial forward primer 8F (5′-AGA GTT TGA TCC TGG CTC AG-3′) and reverse primer 1492R (5′-GGT TAC CTT GTT ACG ACT T-3′). PCR reaction mixture contained 1× Taq reaction buffer, 0.2 μM each primer, 200 μM each dNTP, 30 ng genomic DNA, and 1.5 U Taq DNA polymerase. PCR amplification was carried out by denaturation for 2 min at 95°C followed by 30 cycles of denaturation at 95°C for 45 s, annealing at 45°C for 45 s, extension at 72°C for 1 min, and a final extension step at 72°C for 10 min. The amplicon of 16S rDNA was purified from agarose gel using Gel/PCR DNA Kit (RBC, Taiwan) and sequenced at Bioserve Biotechnologies Pvt., Ltd., Hyderabad, India. The sequence was submitted to GenBank^1^. The isolated strains were identified on the basis of 16S rRNA gene sequence using the EzTaxon server^2^. Phylogenetic tree was constructed using Mega 6.06 software package (Tamura et al., 2013).

### Protease Production and Assay

Isolate S252 was grown in CY broth at 30°C for 5 days with agitation at 180 rpm. The cell-free culture supernatant (CS) was harvested by centrifugation at 8,000 rpm for 15 min and was used as enzyme (PyCP) for further characterization.

Proteases produced by isolate S252 was assayed using 1% casein as a substrate in 0.05 M KCl–NaOH buffer (pH 12). The substrate and enzyme were taken in 1:1 ratio and incubated at 40°C for 1 h. The reaction was stopped by adding twice the reaction volume of 10% trichloroacetic acid (TCA) followed by incubation at 37°C for 30 min. The reaction mix was centrifuged at 10,000 rpm for 10 min and to 500 μl supernatant, 2.5 ml of 0.4 M sodium carbonate was added followed by 250 μl of 1 N Folin-Ciocalteu’s phenol reagent (Meyers and Ahearn, 1977). The reaction was developed at 37°C for 30 min, and absorbance was measured at 660 nm. One unit of protease activity was defined as the amount of enzyme necessary for the release of 1 μg of tyrosine/min/ml under standard conditions. Tyrosine standard curve was generated using L-tyrosine (2–200 μg/ml).

### Sodium Dodecyl Sulphate-PAGE and Zymography

Molecular weight of the extracellular proteases was estimated using 10% sodium dodecyl sulphate (SDS)-PAGE (Laemmli, 1970). The protein bands were visualized by staining with Blue silver stain (Candiano et al., 2004).

The zymogram analysis of the PyCP was performed according to Heussen and Dowdle (1980) with minor modifications. SDS-PAGE was performed by incorporating 0.01% casein in the gel. After electrophoresis, the gel was soaked in 1% Triton X-100 for 1 h, washed in water, and incubated overnight in 0.1 M KCl-NaOH buffer (pH 12). The zone of clearance was observed around the active protease bands.

### Characterization of Alkaline Protease

#### Effect of pH on Activity and Stability of Proteases Produced by Isolate S252

The effect of pH on PyCP was investigated at pH ranging from 8.0 to 13.0. The substrate, 1% casein, prepared in different buffer systems, i.e., phosphate buffer (pH 7), Tris–HCl (pH 8), glycine–NaOH (pH 9 and 10), sodium bicarbonate buffer (pH 11), and KCl–NaOH buffer (pH 12 to 13) was used for the enzyme assay.

The stability of the enzymes was investigated by pre-incubating PyCP in buffers of pH 4.0–12.0 [sodium acetate buffer (pH 4–5), phosphate buffer (pH 6–7)] for 16 h at 4°C or in the buffers of pH 7–12 for 1 h at 40°C followed by determination of residual activity by protease assay.

#### Effect of Temperature on Activity and Stability of Proteases Produced by Isolate S252

The effect of temperature on the activity of PyCP was assessed by carrying out the reaction at 10–70°C for 1 h at optimum pH. The thermostability was determined by pre-incubating PyCP from 40 to 80°C for different time duration (15, 30, 45, 60, 90, and 120 min), and residual activity was measured using standard assay conditions. The enzyme without heat treatment was considered control (100% activity).

#### Effect of Metal Ions

Proteases produced by isolate S252 was pre-incubated with Cu^2+^, Ca^2+^, Mg^2+^, Zn^2+^, Co^2+^, Ni^2+^, Na^+^, and Ag^+^ at different concentrations of 1, 2, and 5 mM for 60 min at 40°C, and residual activity was determined under standard assay conditions. The activity of the enzyme without the added metal ions was considered control (100% activity).

#### Effect of Inhibitors

Proteases produced by isolate S252 was pre-incubated for 60 min at 40°C in 1 mM, 2.5 mM of ethylenediaminetetraacetic acid (EDTA), phenylmethylsulfonyl fluoride (PMSF), aprotonin, and 10 mM of β-mercaptoethanol (β-ME), and the residual activity was measured under standard assay conditions. The reaction mix without inhibitor was used as control (100% activity).

#### Effect of Surfactants and Commercial Detergents

The effect of surfactants on PyCP was assessed using 0.1, 0.5, and 1% (*w*/*v*) sodium dodecyl sulfate (SDS) and 1, 2.5, and 5% (*v*/*v*) Triton X-100 and Tween-20. The effect of commercial solid detergents (0.7%, *w*/*v*); Surf excel, Ariel, Tide, Vanish Oxi Action, and liquid detergent (1:100) Ezee on PyCP was examined. The constituent enzymes in these detergents were inactivated by boiling the detergent solution for 2 h. PyCP was pre-incubated in the respective surfactant or detergent for 1 h at 40°C, and the residual activity was determined using Folin-Ciocalteu’s phenol protease assay. The residual activity of PyCP without detergent incubated at 40°C for 1 h was considered control (100% activity).

#### Effect of Organic Solvents and Hydrogen Peroxide

Proteases produced by isolate S252 was pre-incubated with organic solvents (25%) of methanol, ethanol, isopropanol, acetone, butanol, chloroform, formaldehyde, and formamide for 1 h at 40°C followed by protease assay under standard assay conditions. PyCP was incubated with hydrogen peroxide (H_2_O_2_) (1, 2.5, and 5%) for 40°C for 1 h, and the residual activity was determined using Folin-Ciocalteu’s phenol protease assay. The activity of the enzyme without the added components incubated at 40°C for 1 h was considered 100%.

### Substrate Specificity

To examine the substrate specificity of PyCP, the enzyme was assayed using (1%) casein, gelatin, and bovine serum albumin (BSA) as substrates. PyCP was incubated at the optimum temperature and pH with different substrates for 1 h to determine the relative protease activity.

### Performance Evaluation of Proteases Produced by Isolate S252

#### Washing Performance Test

Clean cotton cloth pieces (approximately 3 cm × 4 cm) were stained with tomato ketchup, coffee, and human blood. After drying, the stained cloth pieces were washed with the commercial detergent Vanish (7 mg/ml), heat-treated Vanish (in boiling water for 60 min to inactivate constituent enzymes), inactivated Vanish supplemented with PyCP (100 U/ml) and water. Cloth pieces washed with water and inactivated Vanish served as negative control. Washing was carried out in tubes for 60 min at 40°C, followed by washing with water and drying. Washing performance was evaluated by visual observation and intensity analysis using Image J software. The percentage stain removal (StR%) was calculated as:


$$\mathrm{Stain}\,\mathrm{removal}\,\left(\mathrm{StR}\%\right)=△\,W/△\,W_{\circ }*100$$

where △*W* is the measure of color intensity difference between washed and unwashed cloth and △*W*ₒ is the difference in the color intensity between clean cloth and the cloth before washing (Hammami et al., 2018).

#### Gelatin Hydrolysis From Waste X-Ray Films

Waste X-ray films were washed with water, wiped with ethanol, and dried at 37°C. The film was cut into pieces, and 900 mg film (pieces) was incubated with 100 U/ml PyCP in Tris–Cl buffer pH 8 and KCl–NaOH buffer pH 12 with continuous shaking at 40°C. The film incubated only in buffers serves as control. The progress of hydrolysis was assessed by measuring the turbidity of the reaction mix at 660 nm till constant turbidity value was observed. The films were also visually examined after complete hydrolysis (Cavello et al., 2013).

#### Dehairing of Goat Skin

Dehairing property of proteases from PyCP was studied using goat skin pieces (3 cm^2^). Goat skin pieces were washed with water to remove extraneous matter and incubated with 200 U/ml of PyCP at 40°C for 24 h with continuous shaking at 150 rpm. Skin pieces incubated with CY broth under same conditions were used as control. After incubation, the skin pieces were dehaired by rubbing and washing in flowing water.

#### Bioextraction of Chitin From Shrimp and Crab Shell

To study the deproteinization of shrimp/crab shells by the alkaline protease, 1 g of shrimp and crab shells washed with water and ground to small particles using pestle and mortar, were treated with PyCP at 40°C. The enzyme was inactivated by heating the reaction mixture at 65°C for 1 h. Subsequently, the shells were washed with distilled water and dried overnight at 50°C. The protein content in the shells was analyzed before and after protease treatment using (FLASH 2000) CHN Elemental Analyzer, Thermo Scientific, United States (CIL Panjab University, Chandigarh, India) to determine the extent of deproteinization. Deproteinization percentage (DP%) was calculated using 6.25 as nitrogen-to-protein conversion factor (Finke, 2013) using the following equation as described by Rao et al. (2000).


$$\mathrm{DP}\%=\frac{\text{Pc}*C-\text{Pt}*T}{\text{Pc}*C}*100$$

where, Pc and Pt are the protein concentrations (%) before and after hydrolysis, while *C* and *T* are the mass in grams of the original sample and hydrolyzed residue on a dry weight basis, respectively. Rate of deproteinization at pH 8 and 12 was also determined by evaluating the protein content in the hydrolysate at different time points using Bradford protein estimation assay.

#### Fourier Transform Infrared Spectroscopy

Fourier transform infrared spectroscopy (FTIR) of chitin was determined using Perkin Elmer Spectrum RX-IFTIR, Canada, spectrometer facility at Sophisticated Analytical Instrumentation Facility (SAIF) at Panjab University Chandigarh, India. The sample was prepared as KBr pellet and processed at room temperature. Pellets were scanned at room temperature (25°C) in the range of 4,000 to 250 cm^–1^ with resolution of 1 cm^–1^ (Hamdi et al., 2017).

#### Scanning Electron Microscopy Analysis

The surface characteristics of the deproteinized shrimp/crab shell samples were observed under scanning electron microscope (SEM) JSM-6100 Jeol, Japan at 50×, 250×, 1,000×, and 1,500× magnifications. The samples were fixed on a sample holder, dried, and sputter-coated gold. Surface regularity was observed and compared for the test and control samples to deduce the effect of deproteinization on shrimp/crab shells.

### Statistical Analysis

All experiments were carried out in triplicate, and data are presented as mean ± standard error. Student’s *t*-test was used to assess the significance of data.

## Results

### Identification of Protease-Producing Myxobacterium

The newly isolated strains of myxobacteria (S236, S244, S250, and S252) along with the previously reported strains were identified as protease producers based on the zone of clearance on skim milk agar medium at pH 11.0 (Kumar et al., 2017). All the tested myxobacterial strains produced extracellular proteases. Based on the highest ratio of zone of clearance/colony diameter, strain S252 was selected for further studies (Table 1).

**TABLE 1:** Protease activity of different isolates.

SNo.	Isolates Id (GenBank accession no.)	Z/C ratio
**1**	S104 (JX316830)	1.89
**2**	S145 (JX316838)	1.43
**3**	S172 (KJ152124)	2.33
**4**	S199 (KM25773)	2.38
**5**	S213 (KM257735)	1.2
**6**	S223 (KP178626)	2.29
**7**	S225 (KM978082)	2
**8**	S229 (KM978086)	1.72
**9**	S233 (KM978088)	2.29
**10**	S236 (KT983630)	1.67
**11**	S244 (KT983635)	1.54
**12**	S252 (KT983642)	3.67
**13**	S250 (KT983640)	2.0

*Z: Diameter of zone of clearance.*

*C: Diameter of the colony.*

*Bold values highlight strain S252 and deproteinization activity of protease produced by S252 (PyCP).*

**FIGURE 1:**
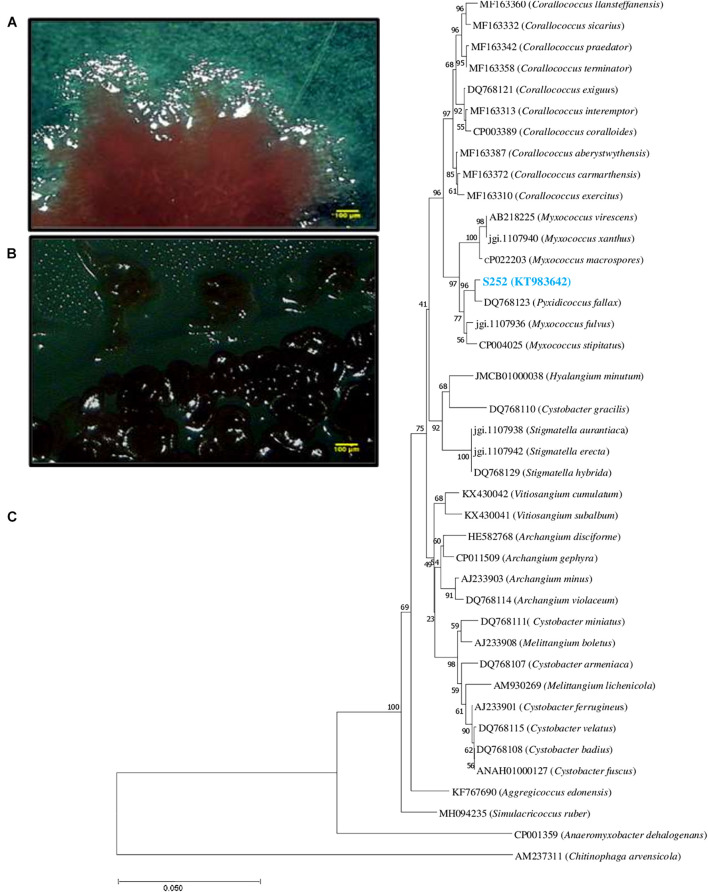
**(A)** Swarm morphology **(B)** fruiting bodies of strain S252 under stereomicroscope. **(C)** Phylogenetic tree based on 16S rDNA gene sequence of *Pyxidicoccus* sp. S252 (blue color text) constructed using neighbor-joining method aligned with the closest type strains.

Isolate S252 is a Gram-negative, swarming bacterium with the ability to produce fruiting bodies (Figures 1A,B). Isolate S252 was identified on the basis of 16S rRNA gene sequence (GenBank accession No. KT983642) using EzTaxon and was found closely related to *Pyxidicoccus fallax* DSM 14698^T^ with 99.57% sequence similarity. The phylogenetic relationship between *Pyxidicoccus* sp. S252 and other myxobacteria in the order Myxococcales is given in Figure 1C.

### Time Course of Protease Production and Zymography

**FIGURE 2:**
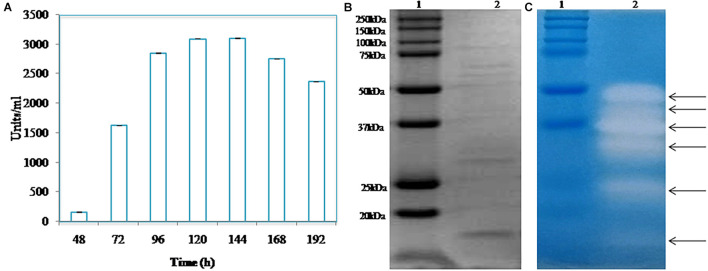
**(A)** Time course of protease produced by isolate S252 after inoculation. **(B)** SDS-PAGE analysis of the extracellular protease. **(C)** Zymogram of caseinolytic activity of extracellular enzyme extract [lane 1, molecular weight marker; lane 2, cell free culture supernatant (CS)].

The optimum temperature of the growth of *Pyxidicoccus* sp. S252 is 30°C. A typical time course of the production of protease by *Pyxidicoccus* sp. S252 at the optimum growth temperature in CY broth is shown in Figure 2A. The extracellular protease production could be detected after 48 h of inoculation which reached a maximum (3000 U/ml) after 120 h of incubation, and the maximum activity was retained till 144 h. SDS-PAGE and zymogram of the extracellular enzyme revealed six proteases with apparent molecular mass ranging from 15 to 50 kDa (Figures 2B,C).

### Biochemical Characterization of Proteases (Proteases Produced by Isolate S252) From *Pyxidicoccus* sp. S252

#### Effect of pH on the Activity and Stability of Proteases Produced by Isolate S252

**FIGURE 3:**
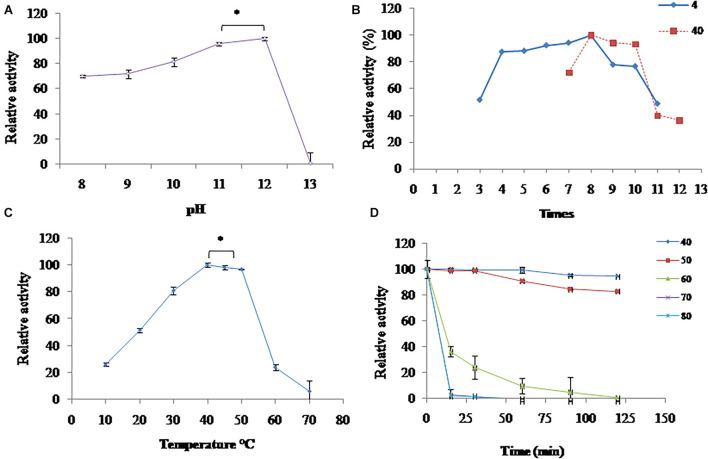
Characterization of extracellular proteases: **(A)** pH optimum; **(B)** pH stability; **(C)** temperature optimum; and **(D)** temperature stability. *Significant difference.

Proteases produced by isolate S252 was highly active in the broad range of pH 8.0–12.0 with maximum activity at pH 12.0 (Figure 3A). At pH 9, 10, and 11, relative activity of 72, 82, and 96% was observed, respectively; whereas at pH 13.0, there was a sharp decline in the activity. The pH stability of PyCP was examined by incubating the enzyme at 4 and 40°C for 16 and 1 h, respectively, in the buffers of different pH values. PyCP was maximally stable at pH 8.0 and retained over 77% of the activity after incubation for 16 h at 4°C over a wide pH range (4.0–10.0) (Figure 3B). When incubated at 40°C for 1 h, the maximum activity (100%) of the PyCP was observed at pH 8.0, followed by 94 and 93% residual activity at pH 9.0 and 10.0, respectively, whereas, relative activity of 40 and 36% was observed at pH 11.0 and 12.0, respectively.

#### Effect of Temperature on the Activity and Stability of Proteases Produced by Isolate S252

Proteases produced by isolate S252 was highly active in the temperature range of 30–50°C and showed a maximum activity between 40 and 50°C (Figure 3C). At 10, 20, and 30°C, PyCP showed 26, 51, and 81% relative activity, respectively.

Proteases produced by isolate S252 was highly stable at 40 and 50°C as it retained 100% activity after 30 min of incubation. After 120 min of incubation at 40 and 50°C, 95 and 83% of the initial activity could be retained, respectively, indicating the thermostable nature of the protease (Figure 3D). The activity of the extracellular enzymes decreased significantly above 50°C and the residual activity of 36% was observed after incubation of the extracellular enzyme at 60°C for 15 min.

#### Effect of Metal Ions and Inhibitors on Proteases Produced by Isolate S252

The effect of various metal ions on PyCP activity was examined (Table 2). An increase in PyCP activity to about 180% was observed in the presence of 1 mM Cu^2+^, 2 mM Na^+^, and 5 mM Na^+^. The addition of 2 mM Mg^2+^ and 1 mM Co^2+^ increased the relative activity of the enzyme to 166 and 115%, respectively. Furthermore, the relative activity showed a slight decrease to 94 and 85% in the presence of 1 mM Ag^+^ and 1 mM Ca^2+^, respectively.

**TABLE 2:** Effect of metal ions and inhibitors on activity of PyCP.

Metal ions	Concentration	Residual activity (%)
Control		100 ± 0.3
Na^+^	1 mM	114.07 ± 1.2
	2 mM	182.79 ± 2.5
	5 mM	179.21 ± 2.8
Mg^2+^	1 mM	105.25 ± 5.6
	2 mM	165.90 ± 3.0
	5 mM	145.85 ± 2.6
Ag^+^	1 mM	94.22 ± 3.1
	2 mM	93.46 ± 0.4
	5 mM	86.22 ± 3.8
Ca^2+^	1 mM	85.05 ± 0.8
	2 mM	82.43 ± 4.0
	5 mM	64.92 ± 7.9
Zn^2+^	1 mM	51.89 ± 0.6
	2 mM	41.61 ± 1.2
	5 mM	22.77 ± 3.0
Cu^2+^	1 mM	180.05 ± 5.5
	2 mM	155.70 ± 0.3
	5 mM	141.64 ± 1.6
Co^2+^	1 mM	114.86 ± 0.8
	2 mM	101.89 ± 2.9
	5 mM	101.13 ± 2.2
**Inhibitors**		
PMSF	1 mM	100.15 ± 1.5
	2.5 mM	109.67 ± 8.7
Aprotonin	1 mM	96.23 ± 0.6
	2.5 mM	93.25 ± 2.6
EDTA	1 mM	97.40 ± 3.7
	2.5 mM	107.60 ± 2.1
β-mercaptoethanol	10 mM	5.0 ± 3.3

*PyCP was incubated with different concentration of metal ions and inhibitors for 60 min at 40°C and the remaining activity was measured under standard assay conditions.*

Phenylmethylsulfonyl fluoride (serine protease inhibitor) and EDTA (metalloprotease inhibitor) did not exhibit any significant effect on activity of PyCP at the concentration of 1 and 2.5 mM, respectively (Table 2). Furthermore, in the presence of 1 and 2.5 mM aprotonin (cysteine protease inhibitor), the enzyme did not show a significant change in residual activity as compared with the control whereas β-ME completely inhibited the activity of PyCP.

#### Effect of Surfactants and Oxidants on Proteases Produced by Isolate S252

Proteases produced by isolate S252 was highly stable in the presence of Triton X-100 and Tween-20 showing an increase in the relative activity to 106 and 135%, respectively at 2.5% concentration of each detergent (Table 3). PyCP retained 80% activity in 0.1% SDS and ∼57% residual activity in 0.5 and 1.0% SDS. PyCP showed activity of 150, 162, and 119% relative to the control in 1, 2.5, and 5% H_2_O_2_, respectively (Table 3). PyCP was markedly stable in the presence of 1 and 5% glycerol, showing 102 and 120% relative activity, respectively as compared with the control. The enzymes were compatible with PEG 8000 also, retaining 86 and 80% activity when incubated in 1 and 5% PEG 8000, respectively for 60 min at 40°C.

**TABLE 3:** Stability of PyCP in the presence of surfactants, oxidant and polyols.

Additives	Concentration	Residual activity (%)
Glycerol	1%	101.76 ± 2.4
	5%	119.96 ± 2.0
PEG 8000	1%	86.08 ± 1.0
	5%	79.65 ± 2.4
Triton X-100	1%	102 ± 3.1
	2.50%	106.66 ± 5.5
	5%	101.50 ± 7.6
Tween 20	1%	130 ± 3.4
	2.50%	135 ± 7.0
	5%	90 ± 1.6
H_2_O_2_	1%	149.78 ± 2.4
	2.50%	162 ± 4.8
	5%	119 ± 10.8
SDS	0.10%	79.63 ± 9.4
	0.50%	57.20 ± 4.1
	1%	56.78 ± 2.7

*PyCP was incubated with different concentration of various additives for 60 min at 40°C and the remaining activity was measured under standard assay conditions.*

#### Stability of Proteases Produced by Isolate S252 Toward Organic Solvents

Proteases produced by isolate S252 was examined against some commonly used organic solvents (Table 4). PyCP was remarkably stable in formamide (retaining 100% activity), acetone (90% residual activity), and methanol (82% residual activity). PyCP showed moderate inhibition (28%) in ethanol whereas 48% inhibition was observed in isopropanol. Chloroform and butanol had detrimental effect on the activity of PyCP.

**TABLE 4:** Effect of organic solvents on activity of PyCP.

Solvents	Residual activity (25%)
Chloroform	40.01 ± 2.5
Butanol	22.57 ± 11.1
Methanol	82.39 ± 1.1
Ethanol	72.20 ± 2.3
Formaldehyde	8.04 ± 6.8
Acetone	89.39 ± 0.3
Formamide	100.85 ± 0.5
Isopropanol	51.97 ± 2.8

*PyCP was incubated with 25% of the solvent for 60 min at 40°C and the residual activity was measured under standard assay conditions.*

### Substrate Specificity

**FIGURE 4:**
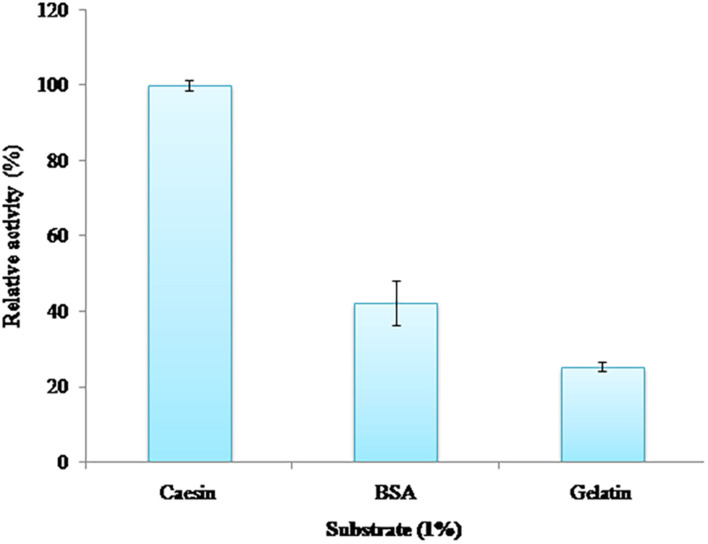
Substrate specificity of PyCP.

Among casein, gelatin, and BSA, casein was the most preferred substrate of PyCP followed by BSA and gelatin, retaining the relative activity of 42 and 24%, respectively (Figure 4).

### Potential of Proteases Produced by Isolate S252 in Detergent Formulations

#### Compatibility With Commercial Detergents

**FIGURE 5:**
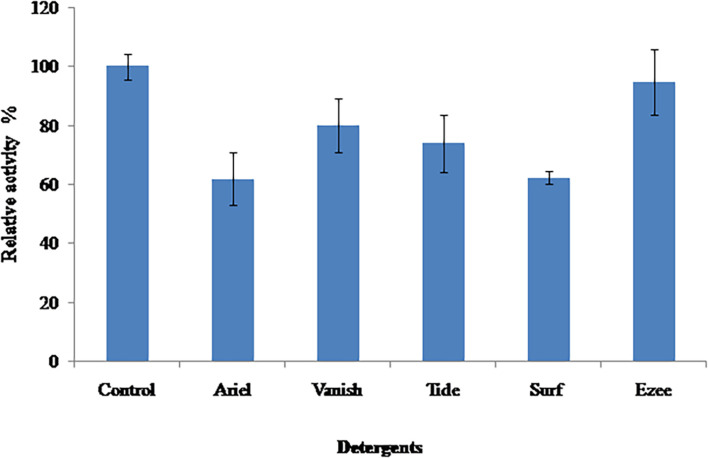
Effect of commercial detergents on stability of PyCP.

The stability of PyCP was evaluated in various commercially available detergents (liquid detergent, 1:100, *v*/*v*; detergent powder, 7 mg/ml) after incubation for 60 min at 40°C (Figure 5). PyCP was extremely stable in liquid detergent Ezee retaining 95% of the initial activity whereas in solid detergents, it was stable in Vanish-Oxi action (containing >30% oxygen based bleaching agent) and Tide showing residual activity of 80 and 74%, respectively. In Ariel and Surf, PyCP lost considerable activity (62% residual activity).

#### Wash Performance Evaluation

**FIGURE 6:**
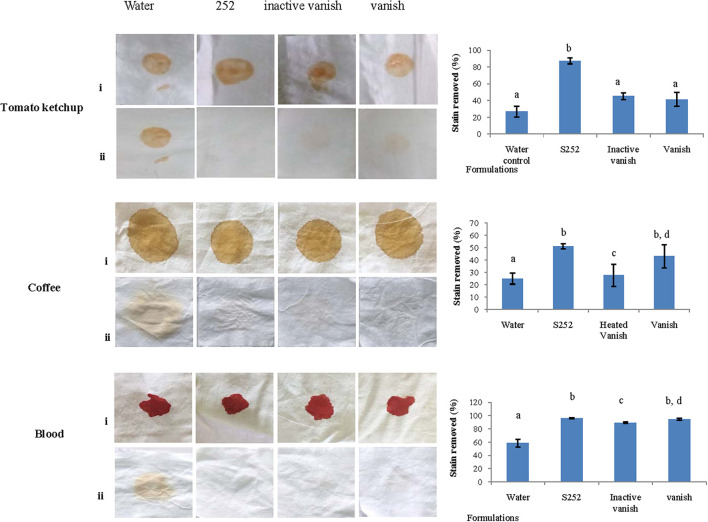
Wash performance analysis of PyCP. Visual observation of stains before and after washing and % stain removal after PyCP treatment is shown. Same letters on the bars represent no significant difference from each other.

As PyCP showed high activity in pH range from 10.0 to 12.0 and was stable in surfactants, ion chelator (EDTA), and oxidant (H_2_O_2_), the application of PyCP was evaluated by assessing its washing performance on clean cotton cloth pieces stained with tomato ketchup, coffee, and blood. The stained cloth pieces were washed with commercial detergent Vanish (7 mg/ml), heat-inactivated Vanish, PyCP in inactivated Vanish and water. PyCP-supplemented inactivated Vanish showed better stain removal ability as compared with water or only Vanish on the stained cloth pieces (Figure 6). Visual appearance of better cleaning was supported by quantitative evaluation where it was evident that PyCP improved the wash performance by 46, 8, and 2% to remove tomato ketchup and coffee and blood stains, respectively as compared with Vanish. Blood-stained cloth pieces showed significantly enhanced wash performance in PyCP-supplemented heated Vanish as compared with heated Vanish whereas no significant difference was detected in wash performance between Vanish and heated Vanish.

#### Gelatin Hydrolysis in Waste X-Ray Film for Silver Recovery

**FIGURE 7:**
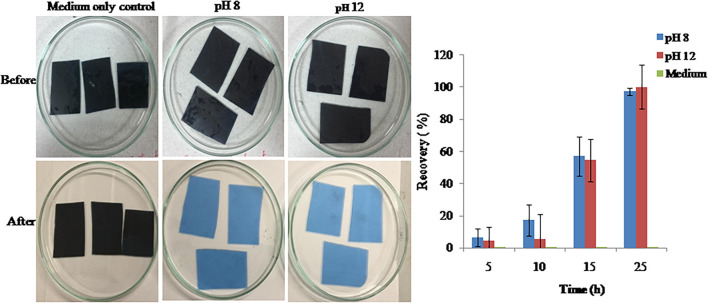
Hydrolysis of gelatin layer from used X-ray films by PyCP.

Proteases produced by isolate S252 can be efficiently used for the recovery of silver from the used X-ray film by hydrolyzing gelatin. At pH 8.0 and pH 12.0, the enzymes showed hydrolysis of gelatin within 25 min leaving a clean polyester sheet while releasing silver into the medium (Figure 7). The turbidity of reaction mix increased till 25 min after which no further increase in the turbidity was observed.

#### Potential of Proteases Produced by Isolate S252 in Dehairing

**FIGURE 8:**
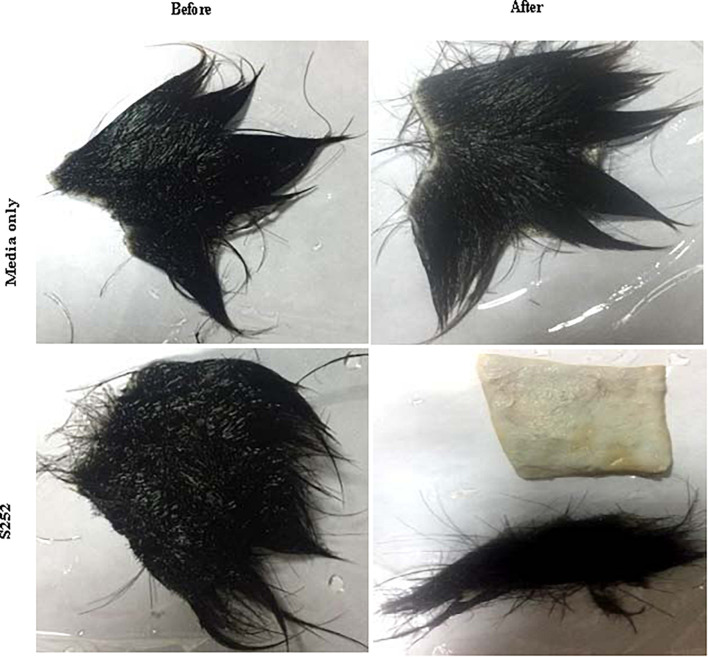
Dehairing potential of PyCP. Goat skin was incubated for 24 h at 40°C with PyCP.

Proteases produced by isolate S252 was evaluated for its potential for dehairing goat skin at the optimum temperature. After the treatment of goat skin with PyCP for 24 h at 40°C, hair could be easily removed from the skin, imparting smooth texture to the surface (Figure 8).

#### Deproteinization of Crustacean Shells for Extraction of Chitin

**FIGURE 9:**
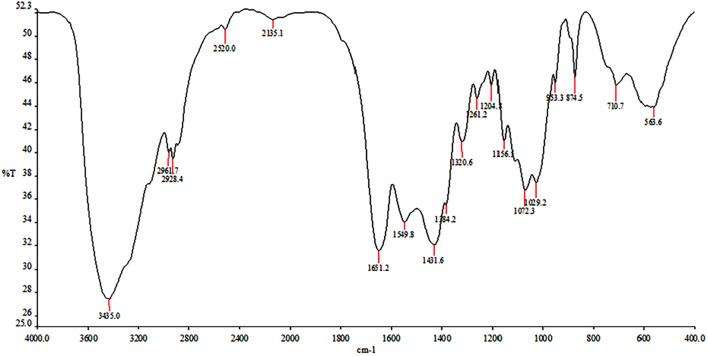
FTIR spectrum of extracted shrimp shell chitin.

**FIGURE 10:**
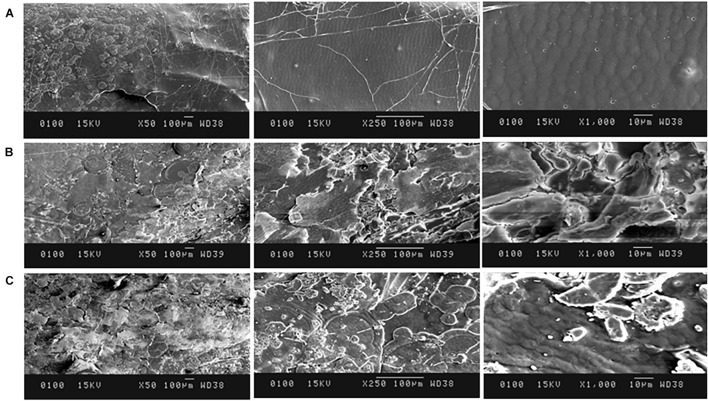
SEM images of shrimp shells **(A)** before treatment, **(B)** PyCP, and **(C)** alcalase.

In crustacean shells, proteins hold the chitin exoskeleton of shrimp shells in a close association making deproteinization crucial in the process of extraction of chitin. The potential of PyCP in extraction of chitin from crustacean shells was evaluated at pH 8 as the enzyme showed maximum stability at pH 8 for 1 h (Figure 3B). The action of PyCP on shrimp shells at enzyme/substrate ratio (E/S) of 20 U/mg resulted in deproteinization of 84% after 6 h (Table 5). In comparison, 20 U/mg papain and alcalase showed 32 and 36% deproteinization of shrimp shells, respectively after 6 h of incubation, highlighting the importance of PyCP in the environment-friendly process of bioextraction of chitin. Extraction of chitin was evident in FTIR analysis of the deproteinized shrimp shell that showed chitin characteristic bands while maintaining the polysaccharide structure (Figure 9). The spectrum obtained for shrimp shell chitin extracted using PyCP showed bands at 1,651, 1,550, and 1,320 cm^–1^ that correspond to the amide I stretching of C = O, the amide II of N–H, and amide III of C–N, respectively. Moreover, IR peaks obtained at 3,435 and 2,932 cm^–1^ is comparable with vibration of −OH and −CO−CH_3_ group, respectively. In addition to this deproteinization of shrimp shell using PyCP showed a significant effect on the surface of shells as evident from the scanning electron micrographs. Before treatment, the shell surface appeared smooth and intact whereas after treatment with PyCP, the surface appeared fractured with abrasions (Figures 10A,B). This suggests that PyCP treatment altered the compact structure of shrimp shells presumably resulting from extraction of protein. The surface of alcalase-treated shells appeared less deformed as compared with PyCP-treated shells (Figure 10).

**TABLE 5:** Deproteinization potential of PyCP.

S. No.	Enzyme	Deproteinization (%)
1	**PyCP**	**84.75** ± 0.67
2	Alcalase*	36.59 ± 3.89
3	Papain**	32.47 ± 6.01

*The data is represented as mean ± standard error of mean of three independent experiments.*

*optimum pH = 8.

**optimum pH = 6.

*Each deproteinization was carried out at E/S of 20 U/mg at 40°C for 6 h. Bold values highlight strain S252 and deproteinization activity of protease produced by S252 (PyCP).*

## Discussion

*Pyxidicoccus* sp. S252, isolated from soil sample, exhibited potent proteolytic activity. The extracellular proteases secreted by the myxobacterial strain S252 were characterized. The limited studies on extracellular proteases secreted by myxobacteria have implicated their role in the life cycle of the host under nutrient-deprived conditions, but their potential applications in the biotechnology applications remain unexplored (Petit and Guespin-Michel, 1992; Dumont et al., 1994). The zymogram of PyCP showed at least six caseinolytic bands ranging from 15 to 50 kDa, suggesting the production of at least six extracellular proteases by isolate S252. The occurrence of extracellular proteases as complexes has been reported in *Myxococcus virescens*, but to the best of our knowledge, there is no report of proteases from *Pyxidicoccus* spp. till date (Gnosspelius, 1978).

The activity and stability of PyCP in the alkaline pH range makes it suitable as an additive in detergent formulations and in dehairing of the skin in the leather industry (Abdel Fattah et al., 2016; Suginta et al., 2016; Champasri et al., 2021; Li et al., 2021). The pH optimum of commercial detergent proteases (Savinase, Subtilisin Carlsberg, and Subtilisin Novo) is pH 10.5, and they are stable in the pH range 7.0–12.0 (Niyonzima and More, 2015). The crude proteases from *Bacillus invictae* were also active in pH range of 9.0–11.0 and could effectively remove blood stains from clothes when added in detergent (Hammami et al., 2017). An alkaline protease from *M. xanthus* (myxobacterium) exhibiting the maximum activity at 40°C and pH 8.5 has been reported, but it was not checked for potential application in the biotechnology industry (Dumont et al., 1994).

The proteases in PyCP had optimal temperatures of 40–50°C and retained more than 80% activity after 120 min at 50°C. Alkaline proteases that are active at temperature around 40–50°C are generally required for detergent industries (Mothe and Sultanpuram, 2016). Alcalase, Savinase, and Maxatase are the commercially used alkaline proteases which have temperature optima between 50 and 60°C (Beg and Gupta, 2003). Thus, PyCP fulfills the desired characteristics of the proteases required in detergent formulations for which high activity at alkaline pH range along with the thermostability is a pre-requisite (Ramkumar et al., 2018). Moreover, PyCP shows >80% relative activity at 30°C, indicating its utility in lower-temperature wash programs making the process energy efficient. It would also be useful in washing of branded garments which are usually not recommended to be washed at higher temperatures (Al-Ghanayem and Joseph, 2020).

It has been well documented that metal ions act as cofactors for proteases, and they either stimulate or inhibit the enzyme catalysis. Several metal ions (Mg^2+^ and Ca^2+^) enhanced the activity of PyCP which further indicated the suitability of the enzymes in laundry detergents for washing in hard water where positively charged Mg^2+^ and Ca^2+^ are present. Several proteases from *Bacillus* spp., Ca^2+^ and Mg^2+^ ions have been reported to stimulate alkaline protease activity (Deng et al., 2010; Annamalai et al., 2014; Yang et al., 2020).

The influence of various protease inhibitors on enzyme activity generally provides insights into the nature of enzyme, active site, and the cofactors required for its activity (Arulmani et al., 2007; Doddapaneni et al., 2009). It was observed that EDTA (metalloprotease inhibitor) did not exert any significant effect on the activity of PyCP, which suggested that PyCP might not contain metalloproteases that require metallic ions for their activity or EDTA could not extract Ca^2+^ bound tightly to the enzymes. The stability of proteases in chelating agents like EDTA make them suitable as an additive in detergents as chelating agents are components of most detergents (Beg and Gupta, 2003). As β-ME completely inhibited the activity of PyCP, it is possible that disulfide bonds are required for maintaining the active conformation of the proteases.

To be used as additive in the detergents, proteases should be compatible with surfactants, oxidants, and other additives. Anionic detergents are most widely used additives in detergent industries because they do not allow the dirt that has been removed from the fabric to reattach to the fabric. In general, proteases are reported to be inhibited in the presence of strong anionic detergent SDS necessitating the requirement for proteases that are stable against SDS (Tatineni et al., 2008; Amid et al., 2014). PyCP showed stability as well as enhanced activity in presence of non-ionic detergents (Triton X-100 and Tween-20), whereas more than 80% residual activity was observed in the presence of 0.1% SDS. The compatibility of PyCP with SDS makes it a promising candidate in the detergent industry where SDS is an unavoidable additive. The crude alkaline proteases from *B. invictae* showed residual activity of 93%, 20% in 0.1, and 0.5% SDS, respectively (Hammami et al., 2017). H_2_O_2_ is a strong oxidant which inactivates the enzymes very rapidly (Yu et al., 2019). PyCP not only showed resistance to a very strong oxidizing agent, H_2_O_2_, but also showed a 62% increase in its activity in its presence. Thus, the resistance of PyCP to H_2_O_2_ reinforces its suitability as an additive in detergents. In literature, very few alkaline proteases have been reported to tolerate H_2_O_2_. The alkaline proteases from *Idiomarina* sp. C9-1 showed enhanced activity of 129 and 126% at 1 and 2% H_2_O_2_, respectively (Zhou et al., 2018). The alkaline proteases from *B. invictae* and *Micromonospora chaiyaphumensis* S103 showed 96 and 78% activity in 1% H_2_O_2_, respectively; whereas the activity dropped to 77 and 37% in 5% H_2_O_2_, respectively (Hammami et al., 2017; Mhamdi et al., 2017). Furthermore, PyCP was also compatible with glycerol and PEG which are used as stabilizers of enzymes in commercial detergents.

The stability of PyCP in formamide, acetone, and methanol indicate the potential of PyCP as biocatalysts in organic synthesis (Doukyu and Ogino, 2010).

To be suitable for various applications, alkaline proteases should have broad substrate specificity. The order of preference of substrates used by PyCP was casein > BSA > gelatin. This suggests, that in addition to the capability of PyCP to remove a wide variety of protein stains from clothes, the enzymes can be also explored for their potential in preparation of casein hydrolysates for use in functional foods and pharmaceutical industry (Tu et al., 2018; Shazly et al., 2019).

The largest share of alkaline proteases is used in laundry detergents as bioadditives because the addition of enzymes confers better washing attributes on the detergent and have the ability to replace some chemicals to make them environmentally compatible (Singh et al., 2018; Zhang et al., 2019). Proteases hydrolyze proteinaceous material in the stain, thus improving the washing efficiency in environmentally compatible, non-phosphate detergents (Maase and van Tilburg, 1983; Jain et al., 2012). Moreover, the use of crude proteases in detergents is economically viable due to the low cost incurred in their preparation as compared with the purified proteases (Paul et al., 2014). The use of alkaline proteases in stain removal from clothes requires the protease to be compatible with ingredients contained in the commercial detergents which adversely affect the stability of the proteases (Reddy et al., 2017). PyCP was highly stable in the liquid detergent Ezee retaining 95% activity. Very few proteases are known to be stable in liquid detergents (Zhang et al., 2019). The increase in preference for liquid detergents among consumers makes PyCP a promising candidate as supplement in liquid detergents. In addition to liquid detergent, the stability of PyCP in the commercial detergent powders (Tide and Vanish-Oxi) suggests the utility of PyCP as an additive in powder detergents also. Several studies have reported variable stability and compatibility of alkaline proteases produced by diverse microorganisms in different commercial detergents (Banik and Prakash, 2004; Abidi et al., 2011; Abdel Wahab and Ahmed, 2018; Ibrahim et al., 2019). Further PyCP was evaluated for washing performance wherein PyCP showed enhanced washing performance against different stains (ketchup, coffee, and blood) as compared with the commercially available detergent Vanish-Oxi. The discoloration of blood stains was obtained by using proteases from *Penicillium chrysogenum* and *Neocosmospora* sp. N1 (Benmrad et al., 2018; Matkawala et al., 2019).

The potential applications of PyCP in hydrolysis of gelatin in X-ray film for silver recovery, hide dehairing and deproteinization of sea waste were also assessed. The exposed X-ray films contribute to 18–20% of silver production worldwide. The conventional method to recover silver from the used X-ray films involves burning the film which results in air pollution (Singh and Bajaj, 2017). Other methods using chemicals have also been developed to extract silver from films which are hazardous to the environment (Zhouxiang et al., 2008). The use of alkaline proteases to remove silver containing gelatin is an environmental friendly option (Masui et al., 2004). PyCP can be efficiently used for the recovery of silver from the used X-ray film by hydrolyzing gelatin. This allows the polyester sheet thus obtained to be reused for manufacturing X-ray film or put into other uses. Silver recovery from X-ray film in 120 min has been recently reported by Thomas et al. (2020).

In the leather industry, animal skins are subjected to treatment with lime and sodium sulfide for dehairing. These toxic chemicals are released as effluents into the water bodies resulting in environmental pollution (Thanikaivelan et al., 2004; Sivasubramanian et al., 2008). There is an increasing shift and interest toward adopting enzymatic processes in leather industry to reduce pollution and also to improve leather quality. Leather industries require lot of water and the physicochemical properties of water would vary depending on season and source (George et al., 2014). PyCP fulfills the requirement of the enzyme that is active and stable in a wide temperature and pH range making it suitable for use in the leather industry. PyCP showed promising results by removing hair and resulting in smooth clear skin. Dehairing of skin has been reported by alkaline proteases produced by *Bacillus* sp. and *Vibrio* sp. CA1-1 (George et al., 2014; Chen et al., 2018; Hammami et al., 2018).

Proteases produced by isolate S252-treated (20 U/mg) shrimp shells resulted in deproteinization of 84% after 6 h. Several proteases from *Bacillus* spp., *Aspergillus* sp., and *Vibrio* sp. have shown 65%–76%, deproteinization of shrimp shells after 3 h incubation at 50°C with E/S ratios of 20 U/mg of protein for bioextraction of chitin (Younes et al., 2012). Deproteinization for chitin extraction from shrimp shell was evident in FTIR spectrum, which was comparable with those of chitin extracted from other sources. The spectrum obtained for shrimp shell chitin extracted using PyCP showed bands at 1,651, 1,550, and 1,320 cm^–1^ that were comparable with three significant amide bands at 1,654, 1,560, and 1,310 cm^–1^, which correspond to the amide I stretching of C = O, the amide II of N–H, and amide III of C–N, respectively; moreover, IR peaks obtained at 3,435 and 2,932 cm^–1^ is comparable with vibration of –OH and –CO–CH_3_ group, respectively for shrimp shell chitin (Liu et al., 2012; Zhang et al., 2021). SEM showed surface abrasion in shrimp shell cell surface morphology which is similar surface deformation that was observed after the successive two-step fermentation treatment with *Serratia marcescens* B742 and *Lactobacillus plantarum* ATCC 8014 (Zhang et al., 2012). The potential of PyCP for deproteinization of shrimp shells can be further explored by optimizing the E/S ratio, temperature, pH, and time of incubation for efficient extraction of chitin. The present study reveals the potential of proteases from *Pyxidicoccus* sp. S252 in commercial processes to make them ecofriendly.

## Conclusion

A new myxobacterial strain, identified as *Pyxidicoccus* sp. S252, secreted extracellular proteases which were active over a wide range of pH and temperature. The alkaline PyCP were stable in several detergent components (surfactants, oxidizing agent, and EDTA) and showed a remarkable washing performance when added to the commercial laundry detergent. Moreover, the enzymes showed excellent gelatin hydrolysis activity on X-ray film exhibiting their potential in silver recovery from X-ray films. Isolate S252 proteases also showed potent dehairing of goat skin and potent deproteinization of crustacean shells. Thus, the present study, for the first time, highlights the potential of alkaline proteases produced by *Pyxidicoccus* sp. in multiple industrial processes. Our future studies are aimed at assessment of extracellular enzymes for commercial exploitation and purification of the enzymes to assess their importance in pharmaceutical industry.

## Data Availability Statement

The datasets presented in this study can be found in online repositories. The names of the repository/repositories and accession number(s) can be found below: https://www.ncbi.nlm.nih.gov/genbank/, KT983642.

## Author Contributions

SS designed and carried out the experiments and prepared the first draft of the manuscript. SK isolated the strain and did preliminary screening. RamK conceived the study, supervised the work, and revised the manuscript. RajK analyzed the results and finalized the manuscript.

## Conflict of Interest

The authors declare that the research was conducted in the absence of any commercial or financial relationships that could be construed as a potential conflict of interest.

## Publisher’s Note

All claims expressed in this article are solely those of the authors and do not necessarily represent those of their affiliated organizations, or those of the publisher, the editors and the reviewers. Any product that may be evaluated in this article, or claim that may be made by its manufacturer, is not guaranteed or endorsed by the publisher.

## Footnotes

1
www.ncbi.nlm.nih.gov/genbank/

2
http://www.ezbiocloud.net/eztaxon/identify
